# Clinical Treatment Experience in Severe and Critical COVID-19

**DOI:** 10.1155/2021/9924542

**Published:** 2021-09-30

**Authors:** Mingliang Li, Donglin Zhu, Jianghua Yang, Ling Yan, Zhiyong Xiong, Jiahai Lu, Xiaogang Bi, Yun Xi, Zeliang Chen

**Affiliations:** ^1^Department of General ICU, The Third Affiliated Hospital of Sun Yat-sen University, Guangzhou 510630, China; ^2^Department of Clinical Laboratory, The Third Affiliated Hospital of Sun Yat-sen University, Guangzhou 510630, China; ^3^School of Public Health, Sun Yat-sen University, Guangzhou 510080, China; ^4^Key Laboratory of Livestock Infectious Diseases in Northeast China, Ministry of Education, College of Animal Science and Veterinary Medicine, Shenyang Agricultural University, Liaoning Province, Shenyang 110866, China; ^5^Department of General Surgery, The Third Affiliated Hospital of Sun Yat-sen University, Guangzhou 510630, China

## Abstract

Compared with other deadly diseases, the coronavirus disease 2019 (COVID-19) is highly infectious with a relatively low mortality rate. Although critical cases account for only 5% of cases, the mortality rate for the same is nearly 50%. Therefore, the key to the COVID-19 treatment is to effectively treat severe patients and reduce the transition from severe to critical cases. A retrospective study was carried out to evaluate outcomes of treatment in patients with severe and critical COVID-19 admitted to a COVID-19 special hospital in Wuhan, China. A total of 75 severe and critical COVID-19 patients were admitted and treated with immunomodulation as the main strategy combined with anti-inflammatory therapy and appropriate anticoagulation. Leukocyte levels in patients with 7-14 days of onset to diagnosis were significantly lower than in those with >14 days. Higher levels of globulin and D-dimer and lower lymphocyte levels were found in the older age group (>65 years) than in the middle-aged group (50-64 years). Patients with comorbidity had higher levels of inflammatory indicators. After treatment, 65 (86.67%) patients were cured, 7 (9.33%) had improved, and 3 (4.00%) had died. Median hospitalization duration was 23 days. Fatal cases showed continuously increased levels of globulin, dehydrogenase (LDH), hypersensitive C-reactive protein (hs-CRP), D-dimer, and cytokines during treatment. Time from onset to diagnosis, age, and comorbidity are important influencing factors on treatment effects. The occurrence of immunosuppression, “cytokine storm,” and thrombosis may be an important cause of death in severely infected cases. In conclusion, high cure rate and low mortality suggested that immunomodulation combined with anti-inflammatory therapy and appropriate anticoagulant therapy is a good strategy for treatment of patients with severe and critical COVID-19.

## 1. Introduction

Coronavirus is a common cause of the human cold but has also led to serious respiratory infectious diseases, such as the Middle East respiratory syndrome (MERS) and severe acute respiratory syndrome (SARS) [1]. The recent outbreak of viral pneumonia was caused by a novel coronavirus, SARS-CoV-2, previously named 2019-nCoV [2]. As of May 20, 2020, the emerging coronavirus infection has caused over 4.9 million cases in more than 200 countries and over 320 thousand of death. Among infected individuals, about 14% become severely, and 5% become critically ill with a mortality rate as high as 50% [3]. Therefore, improving the treatment of severe or critical cases and controlling the transition from severe to critical are the key to improving the clinical cure rate and reducing the mortality rate of COVID-19.

The immunologic state of COVID-19 patients is abnormal, characterized by overactivated inflammatory response, innate immune response, and impaired protective and adaptive immune responses [4, 5]. Early COVID-19 studies have suggested that elevated clinical inflammatory markers are significantly associated with the high risks of the development of severe COVID-19 [6, 7]. Cytokine analysis showed that proinflammatory factors such as IL-1*β*, IL-6, GM-CSF, TNF-*α*, and IFN-*γ* were abnormally elevated in severe COVID-19 patients, which is consistent with the clinical results [8, 9]. Clinical studies have found that cytokine storm is emerging as one of the mechanisms leading to severe COVID-19 infection, which is associated with organ injury and poor prognosis [10]. Cytokine storm is a fatal uncontrolled systemic inflammatory response, which can eventually lead to immune exhaustion and even death. Mild cases are characterized by fever, fatigue, headache, rash, joint pain, and myalgia. Patients with severe symptoms show high fever, headache, fatigue, disseminated intravascular coagulation (DIC), shock, multiple organ failure (MOF), and even death [11–13]. Therefore, suppression of cytokine storm is an important way to effectively prevent disease progression and reduce the mortality rate.

In this study, the epidemiologic and clinical data from 75 patients with severe and critical COVID-19 were analyzed retrospectively. The effectiveness of immunomodulation combined with anti-inflammatory, antiviral, and anticoagulant therapy for severely infected patients was evaluated by analyzing the outcome of the treatment.

## 2. Methods

### 2.1. Patients

From February 11 to March 29, 2020, 75 patients with severe and critical COVID-19 were admitted and treated at Tongji Hospital of Huazhong University of Science and Technology (Wuhan, China). Epidemiologic data, clinical symptoms, past medical history, laboratory findings, and treatment of these patients were recorded.

### 2.2. Definition

According to the guideline of the diagnosis and treatment of COVID-19 in severe and critical cases (2th edition, in Chinese) released by the National Health Commission of China, severe cases are confirmed cases meeting one or more of the following: (1) respiratory distress, defined as respiratory rate (RR) ≥ 30 breaths/min, (2) finger vein oxygen saturation ≤ 93% at rest and without oxygen inhalation, and (3) PaO2/FiO2 ≤ 300 mmHG. In addition, although it has not reached the abovementioned criteria for severe diagnosis, the patients who meet the following conditions will also be considered as severe management cases: immunosuppressed population, those whose lung imaging shows that the foci have progressed to more than 50% within 24-48 hours, and patients aged >60 years with serious comorbidities, including hypertension, diabetes, coronary heart disease, malignant tumor, structural lung disease, and pulmonary heart disease, were also defined severe cases. Critical cases are confirmed cases meeting one or more of the following: (1) respiratory failure occurs, and mechanical ventilation is required; (2) shock; (3) other organ failure occurs, and ICU care is required.

### 2.3. Laboratory Measurements

Real-time reverse transcription PCR assay (RT-PCR) was employed for detection of SARS-CoV-2. Respiratory specimens were collected using nasopharynx or oropharynx swabs and sent to the local designated laboratory for SARS-CoV-2 detection. The nucleic acid detection reagent was purchased from BioGerm Medical Biotechnology Co., Ltd (Shanghai, China), and RT-PCR was performed according to the instructions provided by the manufacturer. Laboratory examination of patients included routine blood test, lymphocyte subsets, coagulation function, globulin, lactate dehydrogenase (LDH), high-sensitivity CRP (hs-CRP), D-dimer, hemoglobin (HB), procalcitonin (PCT), serum ferritin, and immunology indicators, including cytokines and globulins. Chest image inspections were conducted for all patients at admission and discharge.

### 2.4. Treatment Strategy

Treatment for patients with COVID-19 included basic treatment, antiviral therapy, immune regulation, anti-inflammatory therapy, antipulmonary consolidation and fibrosis therapy, anti-mixed infection therapy, and anticoagulation. Basic treatment included oxygen therapy, nutritional support, psychological intervention, rehabilitation exercise, and traditional Chinese medicine therapy. Antiviral drugs were mainly abidol (200 mg three times daily, and the course of treatment does not exceed 10 days), followed by chloroquine (body mass > 50 kg: 500 mg twice daily for seven days; body mass < 50 kg: 500 mg twice daily on day 1 and day 2 followed by 500 mg once daily for the next five days), and hydroxychloroquine (400 mg daily for five days). In consideration of the occurrence of mixed bacterial or fungal infection, some antibiotics and antifungal drugs were used in the treatment. The selection of antibiotics could refer to the monitoring of microbial drug resistance in the hospital and empirically select narrow-spectrum or broad-spectrum antibiotics. Immunomodulatory therapy consisted of zadaxin (1.6 mg daily for 5~7 days followed by 1.6 mg twice a week) followed by human immunoglobulin (5-10 g daily for 3~5 days and 20 g daily for patients with rapid progress), convalescent plasma (200 mL daily for 2~3 days) and tocilizumab. The first dose was 4~8 mg/kg, and the recommended dose was 400 mg through the intravenous drip. Tocilizumab was diluted with 100 mL normal saline, and the infusion time was more than 1 h. For patients with fever, if there was still fever within 24 h, an additional dose was given (same as before), and the interval between the two doses should be greater than or equal to 12 h. The cumulative could not be more than two times, and the maximum single could not exceed 800 mg. Ulinastatin (1.2~1.6 million U daily for more than 5 days), a broad-spectrum protease inhibitor, was mainly used for anti-inflammatory treatment, whereas corticosteroids were relatively less used. Antipulmonary and fibrosis drugs were mainly large doses of ambromide (330 mg three times daily through an i.v. drip) and N-acetylcysteine (600 mg per tablet three times daily). Dabigatran (110 mg twice daily) and low molecular weight heparin (4000-6000 U once daily for patients with high-risk level analysis for deep vein thrombosis) were used for anticoagulation in patients with risk of blood hypercoagulability.

### 2.5. Data Analysis

The continuous and categorical variables were presented as median (IQR), number, or percentage. Differences of laboratory indicators were analyzed with Mann-Whitney *U* test, Wilcoxon signed-rank test, *χ*^2^ test, or Fisher's exact test. A two-sided *α* of <0.05 was considered statistically significant. The SPSS software (version 22.0, IBM, Armonk, NY) was used for data analysis, and GraphPad Prism 7.0 (GraphPad Software, San Diego, USA) was used to describe the continuous changes in multiple laboratory indicators.

### 2.6. Study Approval

The study protocol was approved by the Institutional Review Board of the Third Affiliated Hospital of Sun Yat-sen University (Guangzhou, China) and Tongji Hospital, Tongji Medical College, Huazhong University of Science and Technology (Wuhan, China).

## 3. Results

### 3.1. Demographic and Clinical Characteristics of COVID-19 Cases

A total of 75 cases were admitted from February 11 to March 29, 2020, including 62 severe and 13 critical cases (Table 1). Of these patients, 46 (61.00%) were aged ≥65. In total, 48 (64.00%) patients had fever (including 2 without temperature record), of whom 46 had a temperature of >37.3°C. Many patients had respiratory symptoms, including cough (60, 80.00%), sputum production (37, 49.33%), dyspnea (31, 41.33%), and chest tightness (38, 50.67%). As for gastrointestinal symptoms, the most common symptom upon onset of illness was diarrhea (17, 22.67%). Common concomitant symptoms were fatigue (44, 58.67%), muscle pain (15, 20.00%), and headache (14(18.67%)). Most of patients (67, 89.33%) had one or more comorbidities, with the most common being hypertension (35, 46.67%) and diabetes (14, 18.67%). The median duration from symptom onset to first diagnosis/hospitalization was 14 days, and the median duration of hospitalization was 23 days.

### 3.2. Laboratory Findings, Treatment, and Outcomes of COVID-19 Cases

As shown in Table 2, laboratory test results showed that patients with severe infection had leucopenia (12, 16.00%) and lymphopenia (26, 35.14%) and 43 (76.79%) patients had a PCT concentration < 0.1 ng/ml. The median concentration of HB and D-dimer was 126 g/L and 0.5 mg/L, respectively. The concentrations of LDH, hs-CRP, and fibrinogen (FIB) were higher than the upper limit of reference range values in 38 (51.35%), 57 (76.00%), and 31 (44.29%) patients. Patients received treatment of different drugs, with 50 (66.67%) receiving zadaxin, 41 (54.67%) receiving immunoglobulin, 46 (61.33%) receiving ulinastatin, and 62 (82.67%) receiving acetylcysteine. Short-term low-dose corticosteroid drugs were also administered. In total, 65 (86.67%) patients recovered and were discharged, 7 (9.33%) patients improved and were transferred to other hospitals, and 3 (4.00%) died. For the recovered patients, the median hospitalization time was 23 days. The duration of hospitalization of patients with comorbidity was significantly longer than that for patients without comorbidity (data not shown).

### 3.3. Laboratory Indicators and Chest CT Recovery after Treatment

**Figure 1:**
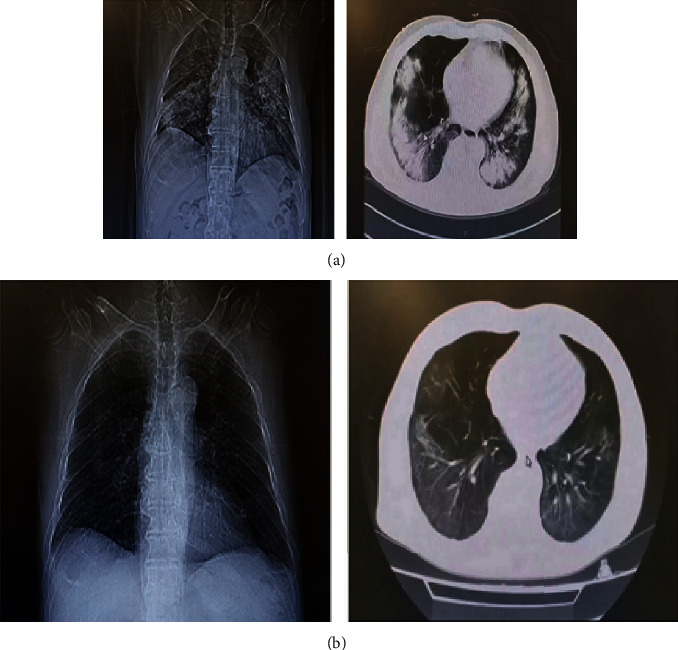
Laboratory indicators and chest imaging of COVID-19 patients before and after treatment. Representative chest imaging at (a) admission and at the (b) end of treatment.

**Figure 2:**
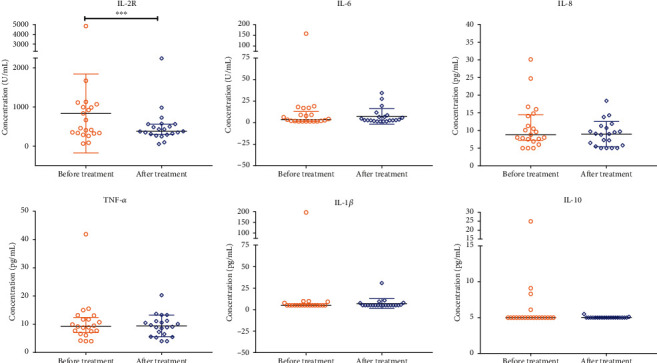
Comparison of cytokine levels in COVID-19 patients before and after treatment. *P* values were tested using Wilcoxon signed-rank test. “∗∗∗” was indicated as *P* value <0.001.

Among the 75 patients, 10 patients without final outcome data (3 died, and 7 were transferred to another hospital) were not included in the analysis. As shown in Table 3, eight indicators improved significantly after treatment, with six indicators (LDH, hs-CRP, D-dimer, FIB, HB, and PCT) decreasing and two (lymphocyte count and lymphocyte ratio) increasing. Chest CT at admission showed multiple ground glass shadows, infiltrative shadows, and consolidation in both lungs but no obvious pleural effusion (Figure 1(a)). Before discharge, chest CT showed multiple ground glass density shadows in both lungs but significantly less consolidation was seen (Figure 1(b)). At admission, the levels of both interleukin-2R (IL-2R) and IL-6 in 13 patients were higher than reference values, and the levels of tumor necrosis factor *α* (TNF-*α*) were increased in 20 patients (Table S1). The level of IL-8 in all the tested patients was undetectable (<5 pg/mL) or normal at admission and before discharge. Only 5 patients showed an increase in IL-2R levels at the end of treatment, and the levels of IL-6 in nearly half of the tested patients were in the normal range. A total of 21 patients were tested for cytokines both before and after treatment. Evaluation of serum cytokines showed the IL-2R levels of patients before discharge were markedly lower than those at admission (Figure 2).

### 3.4. Impact of Diagnosis Timeliness, Age, and Comorbidities on Laboratory Indicators

We divided patients into groups according to time from onset of illness to diagnosis, age, and presence or absence of comorbidities (Table S2). First, the FIB levels of patients whose time from onset to diagnosis was <7 days (<7-day group) were significantly higher than that of patients in the >21-day group. The counts of leukocytes and lymphocytes in the patients in the 7-14-day group were significantly lower than those in the 14-21-day group. Higher levels of LDH and FIB and lower leukocyte counts were found in the 7-14-day group compared with the >21-day group. Compared with patients in the >21-day group, the 14-21-day group showed higher levels of LDH and longer duration of hospitalization. In addition, the older group (>65 years) showed higher levels of globulin and D-dimer but lower levels of lymphocyte count and ratio than the middle-aged group (50-64 years). Finally, patients with comorbidity showed higher levels of globulin, LDH, hs-CRP, D-dimer, FIB, neutrophils, and PCT but lower lymphocyte ratio. The hospitalization time of patients with comorbidity was significantly longer than those without, suggesting that patients with comorbidity have severer illness and need longer time to recovery.

### 3.5. Clinical and Laboratory Characteristics of Fatal Cases

All 3 died cases were male, aged >80 years, and had one or more comorbidities (Table S3). Many indicators in these patients remained at abnormal levels during treatment. As shown in Fig. S1, the globulin level of two patients was over the reference value (>30 g/L) during treatment. The levels of LDH and FIB were increased obviously in patient No. 1. The level of hs-CRP showed an increasing trend in all 3 patients during treatment. The level of D-dimer in patient No. 1 remained relatively low during treatment but began to rise in the several days before death. Blood cell monitoring results showed that the number of neutrophils increased in the late treatment process in three patients whereas lymphocytopenia and decreased HB were found during the entire course of treatment. In addition, the three patients were monitored for the levels of cytokines. The levels of IL-2R, IL-6, and TNF-*α* were significantly higher than reference values. The concentration of IL-1*β* was undetectable on admission but was increased in the middle or late periods of treatment (Table S4). The level of IL-10 in patients No. 1 and No. 3 was dramatically increased in the several days before death (Table S4).

## 4. Discussion

Here, we retrospectively evaluated 75 severe and critical COVID-19 cases and described our experience in the clinical treatment of these patients. Most of the patients were elderly (>65 years old), and their clinical symptoms were consistent with previous reports [14, 15]. In this study, it was observed that fever, gastrointestinal symptoms, and other accompanying symptoms vanished or improved first, and respiratory symptoms improved or disappeared subsequently. The laboratory indicators of severely infected patients have obvious changes at the time of admission.

The elevated inflammation indicators and lymphopenia are considered to be associated with mortality in patients with COVID-19 [6]. Lymphopenia suggests that SARS-CoV-2 may invade lymphocytes as in MERS-CoV, causing lymphocyte apoptosis and damage to the immune system [16]. Lymphocyte count in the older group (aged >65 years) was lower than in other age groups, although no significant difference was found compared with the 25-49-year group, which indicated that the immune system of the elderly patients might be more seriously damaged. In addition, in patients with one or more comorbidities, lymphocyte ratio was significantly lower than in those without comorbidity. Overall higher levels in inflammation indicators and longer duration of hospitalization were observed in patients with comorbidity, which suggests that patients with comorbidity might have higher inflammation levels and disease severity in SARS-CoV-2 infection and need longer time for recovery.

In the absence of specific drugs shown to be effective in the treatment of COVID-19, principles of treatment in severely infected cases are to enhance immunity, improve resistance to virus, and regulate the immune response. In our study, most severely infected patients, especially patients with a low level of globulin or low lymphocyte count, were treated with intravenous immunoglobulins and zadaxin. Zadaxin, also known as thymosin *α* 1, could be used as an adjuvant to improve the immune response of immunosuppressed patients [17]. A previous study showed that the use of zadaxin was able to increase the number of T cells and cytokines in vitro [18]. Less commonly used immunomodulators were convalescent plasma and tocilizumab. Convalescent plasma needs to be provided by rehabilitation patients; as the number of rehabilitation patients grows, the use of plasma has become more extensive [19]. In this study, zadaxin was commonly used in patients with severe and critical COVID-19. Preliminary data show that zadaxin is a safe and effective treatment, which reduces the risk of the transition from severe to critical cases. Tocilizumab is a recombinant humanized monoclonal antibody against the IL-6 receptor [20]. The increased levels of cytokines found in some patients during treatment, including in the three patients who died, suggest that patients may have a cytokine storm, also known as cytokine release syndrome (CRS). There is a clear correlation between the increase of IL-6 in serum and the occurrence of CRS. Therefore, IL-6 has become a potential target for the treatment of CRS caused by SARS-CoV-2 infection. Some studies have suggested that the use of tocilizumab effectively improves patients' symptoms, but more evidence is needed [21–23]. We considered the use of tocilizumab in the treatment of severely infected patients of development stage, especially those with significantly increased IL-6. It is worth noting that the IL-6 levels in nearly half of the tested patients were found to be within normal limits before discharge.

Anti-inflammatory therapy was another treatment that was administered to patients with severe infection, including corticosteroids, mainly dexamethasone and Medrol. Corticosteroids are widely used in the treatment of severe coronavirus infection. However, the safety and efficacy of corticosteroids are still controversial [24]. The use of corticosteroids may be related to prolonged viral clearance, but not in low doses [25–27]. Therefore, the dosage of corticosteroids should be considered carefully. Based on clinical practice, we used doxofylline combined with 5 mg dexamethasone dissolved in 100 ml normal saline for about 5 to 7 days of treatment. If the patient did not need intravenous treatment, we treated with oral 8 mg Medrol once or twice a day. The most widely used anti-inflammatory drug was ulinastatin, which can inhibit the expression of inflammatory factors and oxidative stress, maintain the integrity of lysosomal membrane, and protect tissues and organs [28–30].

In addition to cytokine storm, previous studies have shown that DIC is observed in more than 70% of deaths from SARS-CoV-2 infection [31]. In this study, nearly half of patients had D-dimer levels at admission higher than reference range levels. Patients with the high-risk factors of hypercoagulation or abnormally elevated D-dimer were treated with appropriate anticoagulant therapy, mainly dabigatran and low molecular weight heparin. Another drug commonly used in treatment was acetylcysteine, used for resolving phlegm and preventing pulmonary fibrosis. In the process of treatment, antiviral treatment had no obvious effect; specific drugs for SARS-CoV-2 are needed. During treatment, fewer patients were administered antibiotics because there were fewer cases of mixed bacterial or fungal infections.

Our study shows that treatment with immunomodulation, anti-inflammatory therapy, and appropriate anticoagulation has a curative effect. The 65 patients for whom we had outcome data improved and were discharged, with a cure rate of 87%. Although 13 patients were critical on admission, 8 recovered and 2 improved significantly after treatment. In addition, 62 patients were defined as severe cases on admission, and none of them developed to critical during the treatment. Laboratory indicators and chest CT findings improved significantly before and after treatment. Although immunomodulatory therapy cured the majority of severe patients, three patients died. All three patients were aged >80 years and had one or more comorbidities before they were infected with SARS-CoV-2. Unsurprisingly, there was a sustained decline in lymphocytes in these three patients during treatment, which corresponded to previously released autopsy results for COVID-19 deaths. The autopsy report mentioned that in the patients who died, the spleen was atrophic, the lymph nodes had focal necrosis, and the lymphocyte count was significantly reduced [32, 33]. The increased inflammatory factors seen during treatment indicate that the three patients may have had a strong inflammatory response. The level of IL-1*β* and IL-10 was found to be increased in the middle or late periods of treatment, whereas IL-2R, IL-6, and TNF-*α* were elevated throughout treatment. It is remarkable that IL-8 levels remained normal during treatment in the patients who died and also in the rehabilitation patients, which is different than what is seen in SARS-CoV [34]. The other difference is TNF-*α*, which is not usually elevated in SARS patients [35]. These findings may indicate that there are differences in the immune mechanism and pathogenesis between SARS-CoV and SARS-CoV-2.

There are several shortcomings in this study. First, not all patients received a complete laboratory examination at admission and discharge. Therefore, in the comparative analysis of these laboratory indicators, such as changes before and after treatment, the differences in some indicators may not be reflected. In addition, when comparing the differences in laboratory indicators in different groups, the number of patients in some groups being relatively small, the data analysis results should be viewed cautiously.

## 5. Conclusions

Time to treatment, age, and comorbidity have an impact on treatment effects. Immunosuppression, cytokine storm, and thrombosis may be the important cause of death in severely infected cases. Immunomodulation combined with anti-inflammatory and appropriate anticoagulant therapy improved the cure rate of patients with severe and critical COVID-19. In the absence of specific antiviral drugs, immunomodulatory therapy is able to enhance host immunity and has a good effect on COVID-19 cases with severe infection. Although the infection and immune mechanism of SARS-CoV-2 are still unclear, treatment that addresses immune regulation may improve prognosis.

## Figures and Tables

**Table 1:** Demographic and clinical characteristics of COVID-19 cases upon hospitalization.

Characteristics	All patients (*n* = 75)
Age (IQR), years	66 (59-74)
25-49	12 (16.00%)
50–64	17 (22.67%)
≥65	46 (61.33%)
Male sex	33 (44.00%)
*Clinical classifications*	
Severe cases	62 (82.67%)
Critical cases	13 (17.33%)
*Signs and symptoms*	
Fever	48 (64.00%)
Distribution of temperature	
<37.3°C	9/55 (16.36%)
37.3°C–38.0°C	19/55 (34.55%)
38.1°C–39.0°C	19/55 (34.55%)
>39.0°C	8/55 (14.55%)
Chills	26 (34.67%)
Cough	60 (80.00%)
Sputum production	37 (49.33%)
Dyspnea	31 (41.33%)
Chest tightness	38 (50.67%)
Diarrhea	17 (22.67%)
Nausea	6 (8.00%)
Lack of appetite	43 (57.33%)
Headache	14 (18.67%)
Muscle soreness	15 (20.00%)
Fatigue	44 (58.67%)
Hematuria	1 (1.33%)
*Comorbidity*	
Hypertension	35 (46.67%)
Diabetes	14 (18.67%)
Hepatitis B infection	1 (1.33%)
Cardiovascular disease	7 (9.33%)
Cerebrovascular disease	7 (9.33%)
Chronic obstructive pulmonary	1 (1.33%)
Cancer	2 (2.67%)
Days from onset to diagnosis (IQR), days	14.00 (10.00-23.00)
Duration of hospitalization (IQR), days	23.00 (12.00-32.00)

Note: if denominators of the patients in the analysis differ from the overall number in the group, these denominators are displayed. Percentages may not equal 100 due to rounding. IQR denotes interquartile range.

**Table 2:** Laboratory findings at admission and treatment outcomes.

Laboratory findings	All patients (*n* = 75)
White blood cell count (IQR), ×10^9^/L	5.43 (4.37-7.38)
>10	5/75 (6.67%)
4–10	58/75 (77.33%)
<4	12/75 (16.00%)
Neutrophil count (IQR), ×10^9^/L	3.73 (2.69-4.93)
Lymphocyte count (IQR), ×10^9^/L	1.23 (0.83-1.63)
<1.0	26/74 (35.14%)
≥1.0	48/74 (64.86%)
Procalcitonin (IQR), ng/ml	0.06 (0.05-0.09)
<0.1	43/56 (76.79%)
≥0.1 to <0.25	12/56 (21.43%)
≥0.25 to <0.5	1/56 (1.79%)
≥0.5	0
Hemoglobin (IQR), g/L	126.00 (118.00-134.00)
D-dimer (IQR), mg/L	0.50 (0.30-1.68)
≤0.5	36/72 (50.00%)
>0.5 to ≤1	6/72 (8.33%)
>1	30/72 (41.67%)
Lactate dehydrogenase ≥ 240 U/L	38/74 (51.35%)
Hypersensitive C − reactive protein ≥ 1.0 mg/L	57/75 (76.00%)
Fibrinogen > 4 g/L	31/70 (44.29%)
*Drugs for treatments*	
Zadaxin	50 (67.67%)
Immunoglobulin	41 (54.67%)
Ulinastatin	46 (61.33%)
Acetylcysteine	62 (82.67%)
Dexamethasone	18 (24.00%)
Medrol	5 (6.67%)
*Prognosis*	
Discharge from hospital	65 (86.67%)
Death	3 (4.00%)
Hospitalization	7 (9.33%)
Duration of hospitalization for recovered cases (IQR), days	23.00 (11.00-32.00)
Duration of hospitalization for recovered cases with comorbidity (IQR), days	27.00 (12.50-35.00)
Duration of hospitalization for recovered cases without comorbidity (IQR), days	20.00 (9.50-26.00)

**Table 3:** Comparison of laboratory indicators before and after treatment.

Name	After vs. before treatment					
	*P* value	Ratio	Mean (before)	Mean (after)	Median (before)	Median (after)
GLB, g/L (*n* = 52)	0.5848	99.21%	31.56	31.31	31.70 (28.18-35.70)	31.00 (26.75-35.38)
Lactate dehydrogenase, U/L (*n* = 52)	≦0.0001	60.86%	282.30	171.80	231.00 (201.25-320.75)	169.50 (148.50-187.25)
Hypersensitive C-reactive protein, mg/L (*n* = 54)	0.0004	19.23%	25.21	4.85	8.80 (1.38-22.18)	2.60 (0.80-6.13)
D-dimer, mg/L (*n* = 33)	0.0190	50.91%	1.96	1.00	0.70 (0.31-1.86)	0.47 (0.28-1.19)
Fibrinogen, g/L (*n* = 25)	0.0003	70.07%	6.05	4.24	5.78 (4.26-6.76)	3.74 (3.21-5.45)
White blood cell count, ×10^9^/L (*n* = 57)	1.0000	96.95%	6.10	5.91	5.43 (4.35-7.36)	5.78 (4.82-6.78)
Neutrophil count, ×10^9^/L (*n* = 57)	0.1872	87.25%	4.18	3.65	3.66 (2.74-4.90)	3.35 (2.65-4.29)
Lymphocyte count, ×10^9^/L (*n* = 57)	0.0001	121.00%	1.30	1.57	1.25 (0.82-1.25)	1.52 (1.20-1.98)
Lymphocyte ratio,% (*n* = 57)	0.0018	118.72%	22.86	27.14	22.40 (14.85-22.40)	25.40 (21.10-32.70)
Hemoglobin, g/L (*n* = 56)	0.0001	94.69%	124.20	117.60	123.00 (116.50-131.75)	117.50 (109.00-125.75)
Procalcitonin, ng/ml (*n* = 21)	0.0335	81.61%	0.08	0.07	0.07 (0.05-0.10)	0.06 (0.06-0.07)

*P* value was calculated by Wilcoxon signed-rank test. Data represent median and interquartile range.

## Data Availability

The datasets generated and analyzed during the present study are available from the corresponding author on reasonable request.

## Supplementary Materials

Supplementary Materials Table S1: comparison of cytokine levels in COVID-19 patients before and after treatment. Table S2: difference in laboratory indicators and recovery time among different patient groups. Table S3: demographic and clinical characteristics of fatal cases upon hospitalization. Table S4: changes in laboratory indicators and cytokines during treatment of fatal cases. Fig. S1: tif, changes in laboratory indicators and cytokines during treatment in patients who died. Dynamic change in main laboratory indicator levels (A-I). Dynamic changes of cytokine levels (J-L).
