# Supplementary material for: Clinical Treatment Experience in Severe and Critical COVID-19
Source: Mediators Inflamm. 2021 Sep 30;2021:9924542. doi: 10.1155/2021/9924542 (PMC8483935; doi:10.1155/2021/9924542)
Supplement: Supplementary Materials — Table S1: comparison of cytokine levels in COVID-19 patients before and after treatment. Table S2: difference in laboratory indicators and recovery time among different patient groups. Table S3: demographic and clinical characteristics of fatal cases upon hospitalization. Table S4: changes in laboratory indicators and cytokines during treatment of fatal cases. Fig. S1: tif, changes in laboratory indicators and cytokines during treatment in patients who died. Dynamic change in main laboratory indicator levels (A-I). Dynamic changes of cytokine levels (J-L). [file 9924542.f1.docx]

TableS1. Comparison of cytokine levels in COVID-19 patients before and after treatment.

| **Cytokine** | **Before treatment(n=36)** | **After treatment(n=35)** | ***P*-value** |
| --- | --- | --- | --- |
| **IL-1β,pg/ml** |  |  | 0.7303 |
| <5 | 28(77.78%) | 26(74.29%) | .. |
| >5 | 8(22.22%) | 9(25.71%) | .. |
| **IL-2R,U/ml** |  |  | 0.1004 |
| <223 | 3(8.33%) | 5(14.29%) | .. |
| 223-710 | 20(55.56.%) | 25(71.43%) | .. |
| >710 | 13(36.11%) | 5(14.29%) | .. |
| **IL-6,pg/mL** |  |  | 0.2036 |
| <1.5 | 11(30.56%) | 7(20.00%) | .. |
| 1.5-7 | 12(33.33%) | 19(54.29%) | .. |
| >7 | 13(36.11%) | 9(25.71%) | .. |
| **IL-8,pg/mL** |  |  | 0.3007 |
| <5 | 4(11.11%) | 7(20.00%) | .. |
| 5-62 | 32(88.89%) | 28(80.00%) | .. |
| >62 | 0 | 0 | .. |
| **IL-10,pg/mL** |  |  | 0.9986 |
| <5 | 30(83.33%) | 29(82.86%) | .. |
| 5-9.1 | 5(14.29%) | 5(14.29%) | .. |
| >9.1 | 1(2.86%) | 1(2.86%) | .. |
| **TNF-α,pg/mL** |  |  | 0.8163 |
| <4 | 4(11.11%) | 3(8.57%) | .. |
| 4-8.1 | 12(33.33%) | 10(28.57%) | .. |
| >8.1 | 20(55.56%) | 22(62.86%) | .. |

Each cytokine was grouped according to the normal reference range of cytokine in clinic. P values were tested using χ2 test.

Table S2. Difference in laboratory indicators and recovery time among different patient groups.

| **Name** | **Time interval between onset and diagnosis, days** | | | | | | | | | | | | **Age group,years** | | | | | | **Comorbidity** | |
| --- | --- | --- | --- | --- | --- | --- | --- | --- | --- | --- | --- | --- | --- | --- | --- | --- | --- | --- | --- | --- |
|  | **(<7)vs(7-14)** | | **（<7)vs(14-21)** | | **（<7)vs(>21)** | | **（7-14)vs(14-21)** | | **（7-14)vs(>21)** | | **（14-21)vs(>21)** | | **(>65)vs(25-49)** | | **(>65)vs(50-64)** | | **(50-64)vs(25-49)** | | **（present) vs (not present)** | |
|  | *P* value | Ratio | *P* value | Ratio | *P* value | Ratio | *P* value | Ratio | *P* value | Ratio | *P* value | Ratio | *P* value | Ratio | *P* value | Ratio | *P* value | Ratio | *P* value | Ratio |
| Globulin | 0.8529 | 1.02 | 0.6646 | 0.96 | 0.4451 | 1.06 | 0.2539 | 0.94 | 0.4904 | 1.04 | 0.0832 | 1.11 | 0.0823 | 1.11 | 0.0228 | 1.11 | 0.3051 | 1.00 | 0.0005 | 1.15 |
| LDH | 0.4836 | 0.82 | 0.2569 | 0.74 | 0.3901 | 0.96 | 0.5475 | 0.90 | 0.0358 | 1.16 | 0.0122 | 1.29 | 0.0599 | 1.36 | 0.0525 | 1.24 | 0.5616 | 1.09 | 0.0091 | 1.29 |
| hs-CRP | 0.6886 | 0.56 | 0.8415 | 0.76 | 0.3726 | 2.44 | 0.9195 | 1.37 | 0.1188 | 4.39 | 0.1913 | 3.19 | 0.2612 | 2.56 | 0.2420 | 1.97 | 0.9294 | 1.30 | 0.0001 | 3.64 |
| D-dimer | 0.4305 | 0.72 | 0.0927 | 0.60 | 0.3864 | 0.30 | 0.1562 | 0.83 | 0.7343 | 0.42 | 0.0992 | 0.51 | 0.4844 | 1.47 | 0.0009 | 2.78 | 0.0753 | 0.53 | 0.0087 | 1.91 |
| FIB | 0.5987 | 0.74 | 0.3618 | 0.57 | 0.0162 | 1.91 | 0.5530 | 0.77 | 0.0178 | 2.57 | 0.1239 | 3.35 | 0.1323 | 2.54 | 0.1886 | 2.35 | 0.8317 | 1.08 | 0.0009 | 2.80 |
| WBC | 0.6889 | 1.02 | 0.1251 | 0.78 | 0.1712 | 0.67 | 0.0103 | 0.77 | 0.0104 | 0.66 | 0.9758 | 0.86 | 0.7734 | 0.83 | 0.9815 | 0.85 | 0.8943 | 0.98 | 0.0562 | 1.27 |
| NEUT# | 0.9343 | 0.94 | 0.2432 | 0.71 | 0.1815 | 0.60 | 0.0815 | 0.76 | 0.0525 | 0.64 | 0.8912 | 0.84 | 0.7541 | 0.83 | 0.5126 | 0.90 | 1.0000 | 0.93 | 0.0137 | 1.41 |
| LYMPH# | 0.2964 | 1.19 | 0.6018 | 0.92 | 0.9716 | 0.86 | 0.0212 | 0.77 | 0.1386 | 0.72 | 0.7609 | 0.93 | 0.1790 | 0.83 | 0.0124 | 0.68 | 0.7276 | 1.21 | 0.5088 | 0.97 |
| LYMPH% | 0.3218 | 1.15 | 0.4429 | 1.09 | 0.5870 | 1.17 | 0.9338 | 0.95 | 0.9601 | 1.02 | 0.7609 | 1.07 | 0.2764 | 0.85 | 0.0202 | 0.78 | 0.6099 | 1.09 | 0.0128 | 0.78 |
| HB | 0.3888 | 1.02 | 0.1609 | 1.03 | 0.6553 | 1.01 | 0.3264 | 1.02 | 0.9146 | 0.99 | 0.5132 | 0.98 | 0.8251 | 0.96 | 0.8464 | 0.97 | 0.8091 | 1.00 | 0.1184 | 1.06 |
| PCT | 0.7202 | 0.70 | 0.8615 | 0.73 | 0.4661 | 0.74 | 0.8893 | 1.03 | 0.7514 | 1.06 | 0.8138 | 1.02 | 0.3398 | 1.22 | 0.2107 | 1.37 | 0.9371 | 0.89 | 0.0000 | 1.85 |
| Ferritin | 0.7697 | 0.64 | 0.2482 | 3.01 | 1.0000 | 0.45 | 0.0874 | 4.73 | 0.7815 | 0.71 | 0.0693 | 0.15 | 0.7079 | 0.74 | 0.1763 | 2.43 | 0.7751 | 0.31 | 0.0759 | 1.63 |
| DOH | 0.5215 | 0.81 | 0.0616 | 0.65 | 0.5878 | 1.12 | 0.1453 | 0.80 | 0.0917 | 1.38 | 0.0022 | 1.72 | 0.0985 | 1.37 | 0.0629 | 1.34 | 0.8767 | 1.02 | 0.0119 | 1.40 |

Note: P values were tested using Mann-Whitney U test.

Globulin: Globulin(g/L)

LDH: Lactate Dehydrogenase(U/L)

hs-CRP: Hypersensitive C-reactive protein(mg/L)

D-dimer: D-dimer(mg/L)

FIB: Fibrinogen(g/L)

WBC: White blood cell count(10^9/L)

NEUT#: Neutrophil count(10^9/L)

LYMPH#: Lymphocyte count(10^9/L)

LYMPH%: Lymphocyte ratio(%)

HB: Haemoglobin( g/L)

PCT: Procalcitonin(ng/ml)

Ferritin: Ferritin(ug/L)

DOH: Duration of hospitalization (days)

Table S3. Demographic and clinical characteristics of fatal cases upon hospitalization.

| Number | NO.1 | NO.2 | NO.3 |
| --- | --- | --- | --- |
| Gender | Male | Male | Male |
| Age, years | 88 | 83 | 85 |
| Onset time | 15-Jan | 15-Jan | 1-Feb |
| Time ofdiagnosis | 12-Feb | 11-Feb | 11-Feb |
| Days from onset to diagnosis, days | 28 | 27 | 10 |
| Time of death | 5-Mar | 4-Mar | 28-Feb |
| Duration of hospitalization, days | 22 | 22 | 17 |
| Fever | No |  | Yes |
| Temperature, °C | 36.8 |  | 38.0 |
| Chills | No | No | Yes |
| Cough | Yes | Yes | Yes |
| Sputum production | Yes | Yes | Yes |
| Dyspnea | Yes | No | No |
| Hemoptysis | No | No | No |
| Chest tightness | No | No | No |
| Chest pain | No | No | No |
| Sore throat | No | No | No |
| cyanosis | No | No | No |
| Diarrhoea | No | No | Yes |
| Nausea | No | No | No |
| Vomitting | No | No | No |
| Lack of appetite | Yes | Yes | Yes |
| Abdominal pain | No | No | No |
| Headache | No | No | No |
| Muscle soreness | No | No | Yes |
| Fatigue | Yes | Yes | Yes |
| Night sweat | No | No | No |
| Palpitation | No | No | No |
| Hematuria | No | No | No |
| **Comorbidity** |  |  |  |
| Hypertension | Yes | Yes | Yes |
| Diabetes | Yes | No | No |
| Cardiovascular disease | No | Yes | No |
| Cerebrovascular disease | Yes | No | No |

Table S4. Changes in laboratory indicators and cytokines during treatment of fatal cases.

| **Number** | **Date** | **Globulin** | **LDH** | **hs-CRP** | **D-dimer** | **FIB** | **WBC** | **NEUT#** | **LYMPH#** | **LYMPH%** | **HB** | **PCT** | **Ferritin** | **IL-1β** | **IL-2R** | **IL-6** | **IL-8** | **IL-10** | **TNF-α** |
| --- | --- | --- | --- | --- | --- | --- | --- | --- | --- | --- | --- | --- | --- | --- | --- | --- | --- | --- | --- |
| NO.1 | 12-Feb | 36.1↑ | 210 | 20.2 ↑ | 5.95 ↑ | 5.14 ↑ | 6.77 | 5.16 | 0.83 ↓ | 12.3 ↓ | 141 | 0.08 | 1456.6 ↑ | <5.0 | 1567 ↑ | 10.75 ↑ | 13.8 | <5.0 | 15.2 ↑ |
|  | 16-Feb | 37.4↑ | 244 ↑ | 25.9 ↑ | 2.20 ↑ | 4.69 ↑ | 9.02 | 7.17 ↑ | 0.96 ↓ | 10.6 ↓ | 166 | 0.09 |  | <5.0 | 1524 ↑ | 16.91 ↑ | 23.9 | <5.0 | 15.0 ↑ |
|  | 21-Feb | 31.9 | 195 | 37.5 ↑ | 2.59 ↑ | 4.66 ↑ | 7.85 | 6.40 ↑ | 0.63 ↓ | 8.0 ↓ | 145 | 0.13 | 1578.8 ↑ | <5.0 | 1480 ↑ | 10.76 ↑ | 24.7 | <5.0 | 13.4 ↑ |
|  | 23-Feb |  |  |  | 2.43 ↑ | 3.62 | 5.51 | 5.07 | 0.32 ↓ | 5.8 ↓ | 130 |  |  | 5.2 ↑ | 1400 ↑ | 18.10 ↑ | 20.2 | <5.0 | 27.0 ↑ |
|  | 26-Feb | 34.3 | 225 | 15.1 ↑ |  |  |  |  |  |  |  |  |  |  |  |  |  |  |  |
|  | 29-Feb | 35.5↑ | 223 | 63.1 ↑ |  |  | 6.41 | 5.48 | 0.34 ↓ | 5.3 ↓ | 98 ↓ | 0.13 | 1050.4 ↑ | <5.0 | 1216 ↑ | 33.35 ↑ | 13.7 | <5.0 | 19.1 ↑ |
|  | 3-Mar | 36.0↑ | 288 ↑ | 234.6 ↑ | 2.02 ↑ | 5.91 ↑ | 12.26↑ | 11.50 ↑ | 0.28 ↓ | 2.3 ↓ | 93 ↓ | 3.19 ↑ | 1103.1 ↑ | 7.8 ↑ | 3267 ↑ | 460.60 ↑ | 49.4 | 237.0↑ | 55.3 ↑ |
|  | 4-Mar | 33.3 | 202 | 243.7 ↑ | 3.06 ↑ | 5.04 ↑ | 8.29 | 7.94 ↑ | 0.09 ↓ | 1.1 ↓ | 81 ↓ | 3.65 ↑ |  |  |  |  |  |  |  |
|  | 5-Mar | 35.1↑ | 302 ↑ | 279.1 ↑ | 4.37 ↑ | 5.79 ↑ | 9.83↑ | 9.48 ↑ | 0.05 ↓ | 0.5 ↓ | 114 ↓ | 6.15 ↑ |  |  |  |  |  |  |  |
| NO.2 | 12-Feb | 38.1 ↑ | 305 ↑ | 2.9 ↑ | >21.00 ↑ | 2.96 | 7.90 | 6.16 | 0.78 ↓ | 9.9 ↓ | 170 |  |  |  |  |  |  |  |  |
|  | 13-Feb | 32.7 | 286 ↑ |  | 6.78 ↑ | 4.63 ↑ | 11.87 ↑ | 10.28 ↑ | 0.46 ↓ | 3.9 ↓ | 159 |  |  |  |  |  |  |  |  |
|  | 17-Feb | 21.4 | 256 ↑ | 3.7 ↑ | 14.42 ↑ | 3.64 | 5.48 | 3.61 | 0.76 ↓ | 13.9 ↓ | 131 |  | 653.7 ↑ | <5.0 | 799 ↑ | 61.09 ↑ | 11.6 | <5.0 | 15.0 ↑ |
|  | 22-Feb | 23.1 | 254 ↑ | 19.1 ↑ | 15.99 ↑ | 3.73 | 4.83 | 3.11 | 0.73 ↓ | 15.1 ↓ | 114 ↓ | 0.82 ↑ |  | 7.7 ↑ | 1115 ↑ | 69.96 ↑ | 10.1 | <5.0 | 13.0 ↑ |
|  | 26-Feb | 35.5 ↑ | 257 ↑ | 17.5 ↑ | 13.46 ↑ | 4.37 ↑ | 5.08 | 3.65 | 0.59 ↓ | 11.6 ↓ | 111 ↓ | 1.53 ↑ |  | 8.1 ↑ | 875 ↑ | 175.20 ↑ | 14.1 | <5.0 | 15.3 ↑ |
|  | 29-Feb | 46.5 ↑ | 311 ↑ | 64.2 ↑ |  |  | 6.46 | 5.18 | 0.49 ↓ | 7.6 ↓ | 124↓ | 1.83 ↑ |  |  |  |  |  |  |  |
|  | 3-Mar | 43.0 ↑ | 219 | 99.6 ↑ | 9.07 ↑ | 6.44 ↑ | 11.43 ↑ | 10.69 ↑ | 0.27 ↓ | 2.4 ↓ | 112 ↓ | 4.22 ↑ |  |  |  |  |  |  |  |
| NO.3 | 12-Feb | 38.8 | 315 ↑ | 89.7 ↑ | 1.30 ↑ | 5.20↑ | 5.49 | 3.96 | 1.11 | 20.0 | 136 |  |  |  |  |  |  |  |  |
|  | 17-Feb | 43.1↑ | 364 ↑ | 109.1 ↑ | 5.49 ↑ | 21.20↑ | 9.40 | 8.34 ↑ | 0.64 ↓ | 6.8 ↓ | 133 | 0.16 |  | <5.0 | 1072 ↑ | 138.90 ↑ | 25.5 | 10.2↑ | 12.0 ↑ |
|  | 20-Feb | 43.8↑ | 764 ↑ | 132.7 ↑ | 12.43 ↑ | 53.20↑ | 9.53 ↑ | 8.61 ↑ | 0.53 ↓ | 5.6 ↓ | 133 |  |  | 9.5↑ | 1290 ↑ | 65.05 ↑ | 31.5 | 6.9 | 12.8 ↑ |
|  | 24-Feb | 38.7↑ | 280 ↑ | 85.4 ↑ | 3.21 ↑ | 17.20↑ | 8.80 | 8.39 ↑ | 0.26 ↓ | 3.0 ↓ | 108↓ | 0.24 |  | 9.3 ↑ | 1215 ↑ | 68.33 ↑ | 52.1 | 278.0 ↑ | 25.2 ↑ |
|  | 26-Feb | 45.4↑ | 419 ↑ | 163.5 ↑ |  |  |  |  |  |  |  |  |  |  |  |  |  |  |  |
|  | 27-Feb | 40.4↑ | 373 ↑ | 235.9 ↑ | 6.36 ↑ | 24.10↑ | 16.85 ↑ | 15.56 ↑ | 0.62 ↓ | 3.7 ↓ | 111.0 ↓ | 5.48 ↑ |  |  |  |  |  |  |  |

Note:

Globulin: Globulin(g/L)

LDH: Lactate Dehydrogenase(U/L)

hs-CRP: Hypersensitive C-reactive protein(mg/L)

D-dimer: D-dimer(mg/L)

FIB: Fibrinogen(g/L)

WBC: White blood cell count(10^9/L)

NEUT#: Neutrophil count(10^9/L)

LYMPH#: Lymphocyte count(10^9/L)

LYMPH%: Lymphocyte ratio(%)

HB: Haemoglobin( g/L)

PCT: Procalcitonin(ng/ml)

Ferritin: Ferritin(ug/L)

IL-1β: Interleukin-1β（pg/mL)

IL-2R: Interleukin -2R(U/mL)

IL-6: Interleukin-6（pg/mL)

IL-8: Interleukin-8（pg/mL)

IL-10: Interleukin-10（pg/mL)

TNF-α:Tumor Necrosis Factor-α（pg/mL)

The up arrow indicates that the value exceeds the reference range, and the down arrow indicates that it is below the reference range.

Fig.S1 Changes in laboratory indicators and cytokines during treatment in patients who died.


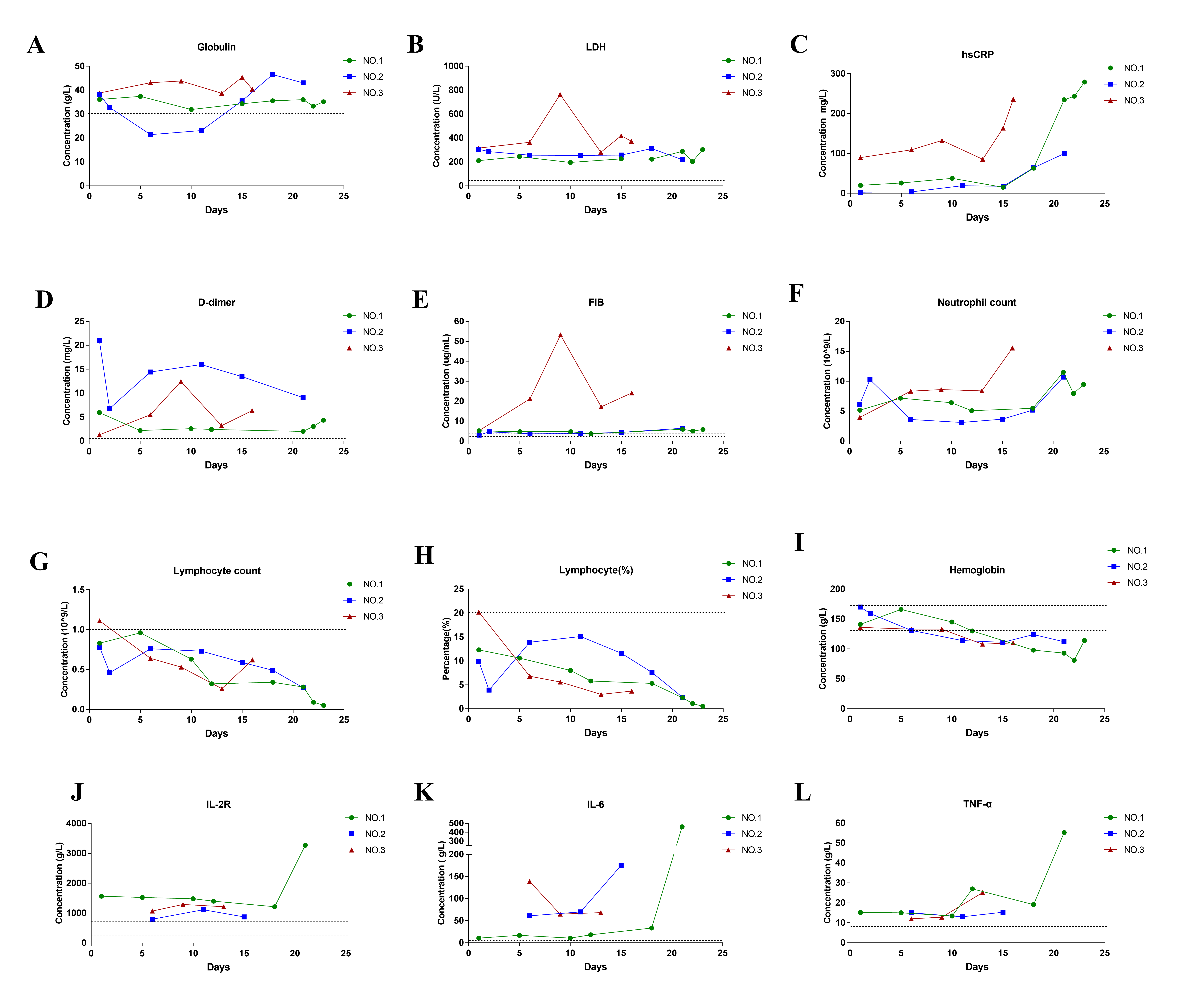


Dynamic change in main laboratory indicator levels (A-I). Dynamic changes of cytokine levels (J-L).
